# Enhanced Adsorptive Properties and Pseudocapacitance of Flexible Polyaniline-Activated Carbon Cloth Composites Synthesized Electrochemically in a Filter-Press Cell

**DOI:** 10.3390/ma12162516

**Published:** 2019-08-07

**Authors:** César Quijada, Larissa Leite-Rosa, Raúl Berenguer, Eva Bou-Belda

**Affiliations:** 1 Departamento de Ingeniería Textil y Papelera, Universitat Politècnica de València. Pza Ferrándiz y Carbonell, Alcoy, E-03801 Alicante, Spain; 2 Departamento de Química Física e Instituto Universitario de Materiales, Universidad de Alicante, Apartado 99, E-03080 Alicante, Spain

**Keywords:** conducting polymer, emeraldine salt state, valence band, flexible composite electrode, dye adsorption kinetics, pseudo-second order model, capacitance, electrical conductivity

## Abstract

Electrochemical polymerization is known to be a suitable route to obtain conducting polymer-carbon composites uniformly covering the carbon support. In this work, we report the application of a filter-press electrochemical cell to polymerize polyaniline (PAni) on the surface of large-sized activated carbon cloth (ACC) by simple galvanostatic electropolymerization of an aniline-containing H_2_SO_4_ electrolyte. Flexible composites with different PAni loadings were synthesized by controlling the treatment time and characterized by means of Scanning Electron microscopy (SEM), X-Ray Photoelectron Spectroscopy (XPS), physical adsorption of gases, thermogravimetric analysis (TGA), cyclic voltammetry and direct current (DC) conductivity measurements. PAni grows first as a thin film mostly deposited inside ACC micro- and mesoporosity. At prolonged electropolymerization time, the amount of deposited PAni rises sharply to form a brittle and porous, thick coating of nanofibrous or nanowire-shaped structures. Composites with low-loading PAni thin films show enhanced specific capacitance, lower sheet resistance and faster adsorption kinetics of Acid Red 27. Instead, thick nanofibrous coatings have a deleterious effect, which is attributed to a dramatic decrease in the specific surface area caused by strong pore blockage and to the occurrence of contact electrical resistance. Our results demonstrate that mass-production restrictions often claimed for electropolymerization can be easily overcome.

## 1. Introduction

Conducting polymers (CPs) are a fascinating family of organic materials that can be easily synthesized with a large diversity of chemical structures and a wide variety of micro- and nano-morphologies, in order to get tailored macroscopic physical and chemical properties [1]. Further, facile and reversible doping enables a set of unique and tunable optical, electronic and redox properties, particularly an electrical conductivity ranging from insulating to metallic. For these reasons, CPs have found promising application as flexible and lightweight functional materials in opto-electronic devices (e.g., light-emitting diodes, thin-film transistors, electrochromic displays, etc.), energy conversion and storage systems (rechargeable batteries, supercapacitors, solar cells or thermoelectric devices), as well as sensor and actuator devices [1,2,3,4]. Owing to the ease of synthesis, low cost, good environmental and chemical stability, high electrical conductivity and capacitance, together with its electrochromic character and ion-exchange properties [5], polyaniline (PAni) has emerged as one of the most industrially important CPs today. These virtues have made PAni an attractive material for a broad spectrum of technologically important applications, such as Li-ion batteries, supercapacitors, electromagnetic interference shielding, electrochemical sensors, and anti-corrosion coatings [1,6]. Also, it is worth mentioning the advent of recent new applications in solid-phase micro-extraction [7] or as (electro)adsorbent/ion-exchange materials for environmental issues [8,9,10], where the affinity of target pollutants for PAni active groups (charged and neutral amine and imine groups) is exploited to an advantage.

However, PAni has the main shortcomings of a relatively low porosity, specific surface area, slow degradation, and poor mechanical stability because of volume changes produced upon repeated charge/discharge process [2,4,11,12]. Composites of PAni with carbon materials, metal oxides, natural and modified clay minerals, zeolites or mesoporous silica [2,4,9,10], have been extensively investigated with the goal of overcoming these disadvantages and further improving existing properties via synergistic effects. Carbons are outstanding versatile materials with regards to their use as composite supports because of the wide availability of allotropes, microtextures, 0 to 3D dimensionality, and macroscopic forms. In addition, their excellent chemical and thermal stability, good electrical conductivity, large specific surface area, wide range of pore structures, and mechanical strength [11,12] make them particularly suitable as composite constituents for electrochemical applications or environmental adsorbents. To date, much work has been undertaken on PAni composites with carbon nanotubes [13,14], graphene/reduced graphene oxide nanosheets [4,13], porous carbon foams or rods [15,16] and activated carbon fibers [11,17] or powders [18,19]. Only recently, activated carbon fiber cloths (ACC) have gained popularity as inexpensive, highly porous and flexible mechanical supports for conducting polymers in electrode materials for wearable power microelectronics or roll-up electrochemical systems [8,20,21,22,23,24,25]. Furthermore, the highly porous 3D conductive framework of the carbon fabric allowed PAni-ACC composites to be directly used as electrodes without the use of insulating binders and conductive additives. Thus, the unique combination of wide accessibility of the fabric pore nanoarchitecture [24]; the binderless CP–carbon interface [25]; and simple, fast and reversible surface redox reactions in PAni [24] has shown to provide the composite with shortened path lengths for direct electron transfer and fast ion transport [20,22,24,25].

PAni-carbon composites have been synthesized by conventional in situ chemical, emulsion/interfacial, vapor chemical or electrochemical polymerization, just to cite a few [6,26,27]. Electrochemical methods have proven to be simple and powerful tools to produce uniform, adherent thin PAni films over a number of different conductive substrates [6], including porous carbon materials, which have served as hard templates capable of transcribing their nanostructure to the growing polymer. However, it is often claimed that electrochemical routes for the preparation of PAni are limited in terms of mass production [23]. In fact, the vast majority of examples of electrosynthesized carbon-PAni composites deal with small size (typically 1 to 2 cm^2^) samples, while research treating significantly larger areas is scarce [8,28] and generally does not focus on a systematic study of the effect of synthesis variables on the structure and properties of the resulting composites.

In this work, we show the feasibility of producing flexible PAni-ACC composites of large size (~20 cm^2^) by simple galvanostatic (i.e., constant current) electrolpolymerization in a filter-press electrolyzer, a type of cell design advantageous for industrial scaling-up [29]. The polymer loading density was controlled by changing the electropolymerization time (i.e., the amount of passed charge), and the surface microstructure, chemical composition, porous texture, and thermal stability of the fabricated composites were studied by Scanning Electron microscopy (SEM), X-Ray Photoelectron Spectroscopy (XPS), N_2_/CO_2_ adsorption experiments, and thermogravimetric analysis (TGA). Some important properties for practical application as electrodes in supercapacitors, secondary batteries or as adsorbent materials in dyestuff effluent treatment were examined and correlated with their structural and chemical features. For this purpose, the capacitance and electrical conductivity were derived from cyclic voltammetry (CV) and four-strip probe conductance experiments, and the liquid-phase adsorptive capability was studied by using aqueous solutions of Acid Red 27, a model dye of anionic azo dyes used in the food and textile industries.

## 2. Materials and Methods

### 2.1. Materials

A viscose-based activated carbon cloth (CTex-20, knitted fabric, areal density: ~18 mg cm^−2^, mean fiber diameter: ~10 μm) was purchased from Mast Carbon International Ltd. (Hampshire, UK). The carbon fabric was cut in sheets of 45 mm × 55 mm. The untreated carbon samples were repeatedly washed with distilled water until constant pH. Then, they were dried in an oven at 80 °C overnight and finally allowed to cool down in a desiccator. Analytical reagent-grade aniline (≥99.0%) and sulfuric acid (98%) were supplied by Merck. Prior to use, aniline was distilled under vacuum and stored in the dark at 5 °C. Solutions of aniline (0.1 M) in aqueous H_2_SO_4_ electrolyte (0.5 M) were made up with distilled water. Background electrolyte solutions (0.5 M H_2_SO_4_) for cyclic voltammetry were prepared from Millipore Milli-Q water. The anionic monoazo dye Acid Red 27 (Colour Index no. 16185, empirical formula C_20_H_11_N_2_Na_3_O_10_S_3_) was purchased from Sigma-Aldrich (91 wt.% dye content) and used as received.

### 2.2. Preparation of Hybrid PAni-ACC Composites

**Figure 1:**
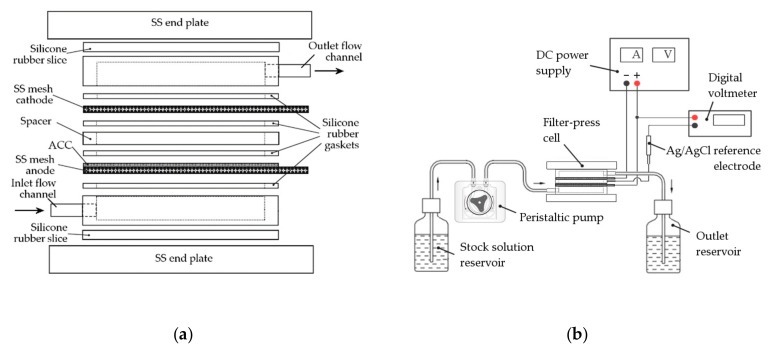
(**a**) Cut-away view of the electrochemical filter-press cell; (**b**) Schematic diagram of the electropolymerization system.

Polyaniline was polymerized on the carbon fabric substrate by galvanostatic treatment in an undivided home-made filter-press electrolyzer, designed for the electrochemical processing of textile-structured materials (Figure 1a) [30,31]. The cell was assembled in a flow-through parallel plate-and-frame configuration, with two identical stainless steel (SS) mesh electrodes separated by a 5-mm-thick plastic spacer, providing an interelectrode rectangular channel of 20 cm^2^. A dry ACC sheet (typically 0.4 g) was pressed against the anode SS mesh with a silicone rubber sealing gasket to leave an exposed geometric area of 20 cm^2^. The aniline-containing electrolyte was fed into the cell with the aid of a peristaltic pump (Dinko Instruments D-21V, Barcelona, Spain), until excess solution was collected at the outlet. As a pre-conditioning step, the system was left at open circuit for 30 min to allow carbon pore filling and facilitate aniline adsorption. Then, an input current of 100 mA (~14 mA cm^−2^ g^−1^) was passed through the cell for different processing times (10–120 min) at room temperature. The anode potential was measured against an Ag/AgCl reference electrode connected through a Luggin capillary drilled in the plastic spacer, and it was found to lie within the range 0.6–0.8 V. A schematic view of the electrochemical set-up is shown in Figure 1b. After the prescribed electropolymerization time, the modified carbon cloth was washed repeatedly with 0.5 M H_2_SO_4_ and subsequently with distilled water. The obtained PAni-ACC composite was dried at 40 °C under dynamic vacuum for 24 h and stored in a desiccator until further characterization studies.

### 2.3. Materials Characterization

The morphology and micro-structure of the unmodified ACC and the PAni-ACC composites were examined by SEM. Secondary electron micrographs were obtained with a Phenom Microscope (FEI Co., Hillsboro, USA). X-Ray Photoelectron spectroscopy (XPS) was conducted in a K-ALPHA spectrometer (ThermoFisher Scientific) by using a microfocused monochromatized Al Kα radiation (1486.6 eV) of 400 μm spot size at 173 K and a base pressure below 5 × 10^−10^ kPa. Photoelectrons were collected into a hemispherical analyzer operated in the constant energy mode at pass energy of 50 eV for narrow core-level and valence band spectra. Peak binding energies (BE) were referenced to the principal C1s line at 284.6 eV and given to an accuracy of ±0.2 eV. XPS data were analyzed with Thermo Scientific^TM^ Avantage software. A smart correction function was used for background correction. Peak synthesis was done with mixed Gaussian (70%)/Lorentzian (30%) function lineshapes. Surface charging was compensated with a flood electron gun.

The porous texture was determined by physical adsorption of N_2_ (at 77 K) and CO_2_ (at 273 K) by using an automatic adsorption system (Autosorb-6, Quantachrome Instruments, Boynton Beach, USA). In order to remove moisture and adsorbed gases while avoiding thermal degradation of PAni, the samples were previously out-gassed under vacuum at 423 K for 4 h. The apparent specific surface area (S_BET_) and total micropore volume (d < 2 nm, V_μ_) were calculated from the N_2_ isotherm by applying the Brunauer-Emmett-Teller (BET) and the Dubinin-Radushkevich (DR) equations, respectively [30]. The DR theory was also applied to obtain the ultramicropore volume (d < 0.7 nm, V_ultra μ_) from the CO_2_ isotherm [30]. The good fitting of the N_2_ and CO_2_ adsorption data to the DR equation (*R*^2^ > 0.99) validated the application of this method for the studied materials. The N_2_ uptake at a relative pressure near to capillary condensation (~0.97) was used to calculate the total pore volume (V_tp_) [32]. The mesopore volume (2 nm < d < 50 nm, V_meso_) was estimated as the difference between total and micropore volumes.

The thermal stability of the samples was studied by thermogravimetric analysis (Mettler Toledo 851E/1600/LG) under a He stream at a flow rate of 100 mL min^−1^. About 10 mg of sample was placed in a 70 μL aluminum crucible and submitted to a two-stage heating protocol at a rate of 20 °C min^−1^. First, the samples were heated from 25 °C to 120 °C and kept at the latter temperature for 30 min for drying. Then, they were allowed to cool down to thermal equilibrium at 25 °C. In the second stage, the samples were heated up to 1000 °C.

### 2.4. Electrochemical and Sheet Resistance Measurements

Cyclic voltammetry (CV) experiments were conducted in an all-Pyrex glass cell with provision for a standard three-electrode assembly. Round-shaped cut, dry ACC and PAni-ACC samples of approximately 1 cm^2^ (10–13 mg) were weighed to an accuracy of ±0.001 mg. The samples were pressed at 20–25 kg cm^−2^ for 40 s in between a folded SS mesh, used as a current collector for the working electrode assembly. Prior to characterization, these electrodes were immersed in 0.5 M H_2_SO_4_ overnight to promote the material impregnation. A Pt wire was employed as a counter electrode. The working electrode potential was given with reference to a commercial Ag/AgCl/Cl^−^ (3.5 M KCl) electrode. The measurements were carried out in a potentiostat system (VSP model, Bio-logic Science Instruments). The working solution was previously de-aerated by bubbling N_2_ and blanketed throughout all the experiments by a N_2_ stream flowing over it. Cyclic voltammograms were recorded at room temperature at a scan rate of 1 mV s^−1^ and presented as mass-normalized current (mA g^−1^) vs. potential plots. The gravimetric specific capacitance was evaluated from CV (Appendix A, Equation (A1)).

The conductivity of ACC and PAni-ACC composite fabrics was measured by the four-strip probe method [33]. Dry sample sheets (11 mm × 40 mm) were sputter coated with four Pd-Au thin strip probes (5 mm × 11 mm) in an EMITECH SC7620 Sputter coater (Quorum Technologies Ltd, Laughton, UK). The sputtered probes were 5 mm equally spaced across the length of the sample sheet. Copper wires were glued to the probes by conducting silver epoxy resin, and the contacts were secured with thin aluminum foils. A constant current, *I*, from a DC power supply (EP-613, Silver electronics) was passed through the two outermost probes and measured with a digital multimeter in series. The voltage drop, *V*, across the two inner probes was measured in a FLUKE 45 dual Display digital voltmeter. The electrical resistance, *R* (Ω), was obtained from the linear slope of *V* vs. *I* plots in the range 0–10 mA. The surface sheet resistance, *R*_s_ (Ω ☐^−1^), was derived from the calculated electrical resistance according to Equation (A2) (see Appendix A) [34].

### 2.5. Dye Adsorption Measurements

A synthetic stock amaranth solution was prepared by dissolving 1 g of dye in 1 L of distilled water. Working solutions of concentration in the range 25–200 mg L^−1^ were obtained by proper dilution with distilled water. Dry ACC or PAni-ACC composites were cut in pieces of about 2 cm^2^ and accurately weighed to ±0.1 mg (typical weights ~0.035–0.04 g). In a typical adsorption experiment, 50 mL of dye solution of known initial concentration were contacted with the adsorbent in 100 mL glass bottles with a screw cap, that were further sealed with Parafilm^®^. The contact was made in a thermostatic water bath shaker (model WNE22, Memmert, Schwabach, Germany) at a constant temperature of 25 °C and at an agitation speed of 120 rpm, for the time necessary to reach equilibrium. After prescribed time intervals, the liquid-phase dye concentration was analyzed in a Thermo Scientific Helios γ UV-vis spectrophotometer at λ_max_ = 520 nm.

## 3. Results

### 3.1. Characterization of Surface Morphology, Surface Chemistry and Porous Structure

#### 3.1.1. Analysis of the Surface Morphology by SEM Imaging

**Figure 2:**
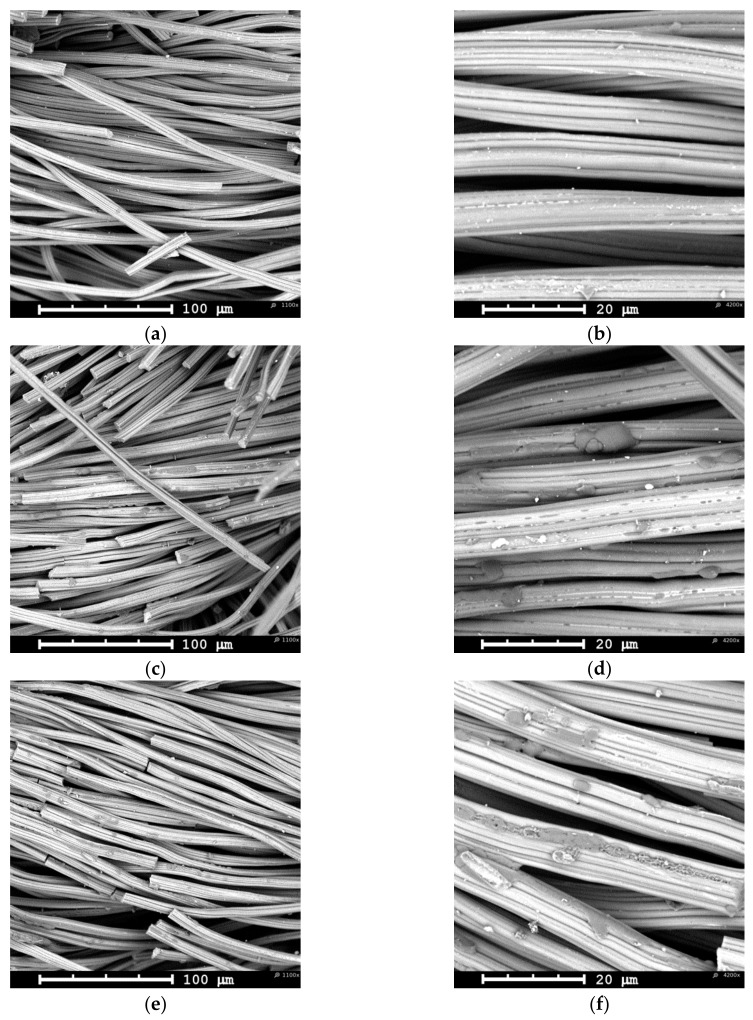
Low- and high-magnification Scanning Electron microscopy (SEM) micrographs of (**a**), (**b**) untreated activated carbon cloth (ACC) and PAni-ACC composites synthesized after (**c**), (**d**) 20 min and (**e**), (**f**) 40 min of electropolymerization.

Figure 2a (low magnification) shows the typical morphology of a yarn in the knitted AC fabric. Each yarn is a twisted bundle of loose carbon fibers of about 10 μm in average diameter. A higher magnification image (Figure 2b) reveals that each fiber has a ridge surface with grooves parallel to the fiber axis direction. This shape is typical of wet spun viscose fibers [35] used as precursor material in the activated carbon fabric manufacture. At an electropolymerization time of 10 min, PAni can already be discerned on the surface of ACC (Figure 2c–d). The polymer appears to be distributed over the carbon fabric in a scattered fashion and in the form of smooth and compact deposits on the surface of the fibers. These deposits are preferentially localized around the grooves in a carbon fiber. The number of such PAni deposits increases slightly with the electropolymerization time, their size seems also to grow along the fiber grooves, and some degree of roughening appears (Figure 2e–f). In all these examples, the polymeric material was only distinguished on the ACC face in contact with the stainless steel anode inside the filter-press cell, whereas no evidence of PAni was found on the opposite face. However, the surface N/C quotient (as measured by XPS, see Section 3.1.2) increases with the increasing time of treatment at both sides of the ACC. The evolution of this ratio is a diagnostic signal that the polymer grows throughout the whole fabric surface, but whenever it does as a very smooth and thin film, it may appear morphologically featureless and barely discernable by SEM at the magnification reached in Figure 2 [36].

**Figure 3:**
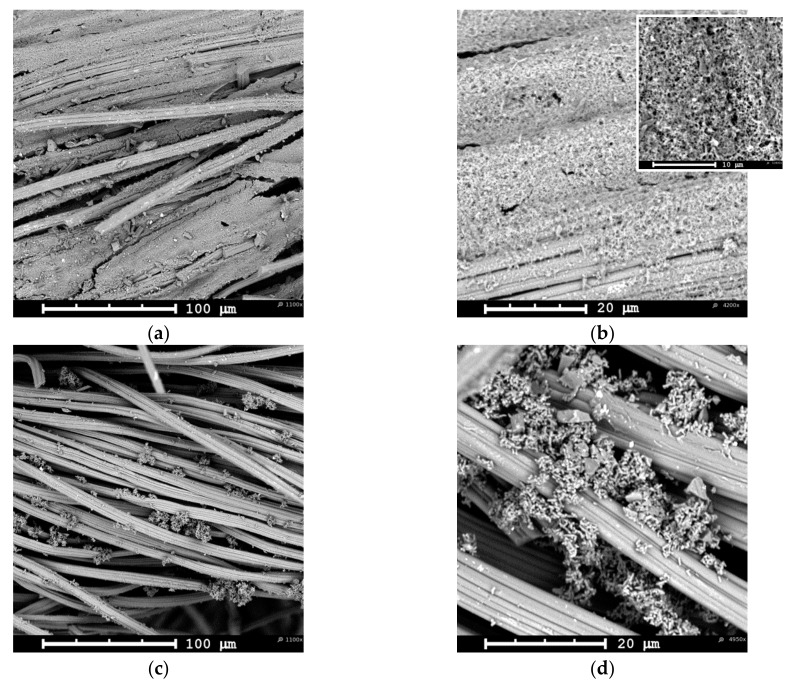
Low- and high-magnification SEM micrographs of PAni-ACC composites synthesized after 120 min of electropolymerization, (**a**), (**b**) electrode side; (**c**), (**d**) electrolyte side.

After 120 min of galvanostatic treatment, a remarkable increase in the amount of electrodeposited conducting polymer is observed (Figure 3). At this stage, a thick PAni layer grows on the ACC face in contact with the anode surface, fully covering most of the carbon fibers and even filling most of the void space among fiber bundles in a yarn (Figure 3a). This thick PAni coating appears brittle and easily peels off upon slight fabric bending. Also, some polymer material detached from the fabric during manipulation for withdrawal from the cell and during subsequent rinsing. High magnification micrographs show that the PAni layer is rather porous (Figure 3b) and can be properly described as a nanofibrous mat (see inset to Figure 3b). Localized open networks of either nanofibrous polyaniline or aggregates of short and randomly aligned nanowires can also be discerned on the face exposed to the electrolyte (Figure 3c–d). These supramolecular structures of polymeric material are unevenly distributed among the individual carbon fibers forming a yarn and their proportion to the carbon fabric is much lower than that in the opposite face of the cloth. Accordingly, the XPS N/C atomic ratio is also lower than that for the side fully coated by a thick polymer layer (see below).

#### 3.1.2. Surface Chemical Composition by XPS.

*(1) Core-Level Spectra*

The surface elemental composition, expressed as atomic percentage, of fresh and PAni-modified ACC at different electropolymerization times, was computed from integrated photoelectron areas and is summarized in Table S1. The small amount of nitrogen-containing surface complexes present in fresh ACC (1.2 at.%) most likely stems from nitrogen existing in the precursor carbon source [37]. Sulfur can also be present in the precursor source as elemental sulfur impurities or in the form of inorganic or organosulfur compounds at low proportion [37]. The oxygen content mostly results from carbon-oxygen surface complexes that develop as chemical defects at the edges of graphene layers.

The nitrogen and sulfur content on the surface increases with the increasing electropolymerization time in connection with the growth of polyaniline (Table S1). Nitrogen is a characteristic constituent of PAni polymeric chains either in the form of amine or imine links between benzenoid or quinonoid moieties in the linear polymer backbone or as charged (protonated) nitrogen connected to semiquinone segments. The increased amount of sulfur is due to electrolyte (bi) sulfate anions incorporated into the polymer structure as the dopant, although some residue from inefficient electrolyte removal by rinsing cannot be ruled out. The analysis of the different bonding states of these elements will be treated in detail further below.

**Figure 4:**
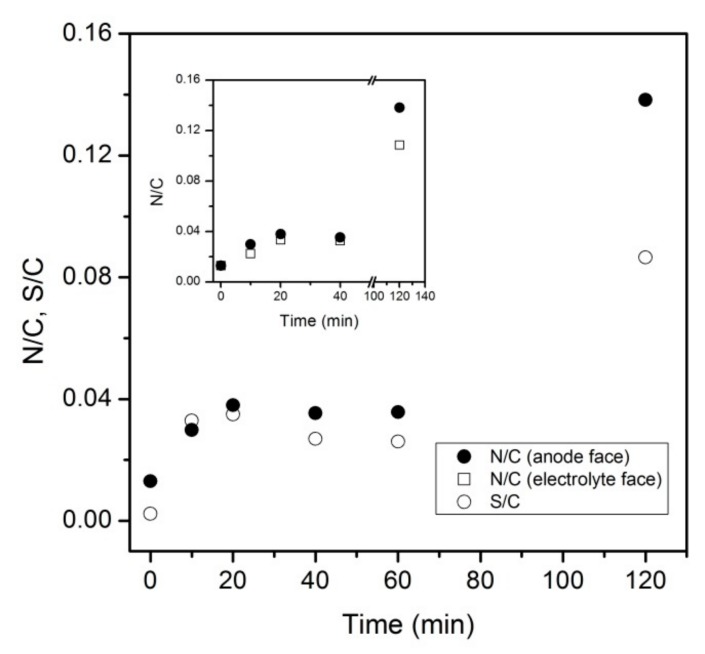
Evolution of surface N/C and S/C ratios for PAni-ACC composites as a function of the electropolymerization time. Inset: Comparison of N/C ratios at both sides of the fabric.

The plot of the N/C and S/C atomic ratios vs. time (Figure 4) shows that the N and S content in the hybrid PAni-ACC composites increases moderately at short times, it seems to level off at intermediate electropolymerization time and eventually rises sharply at prolonged process time (120 min). The value of the N/C ratio at short electropolymerization time is lower than the theoretical one for pure linear polyaniline chains (N/C_PAni_ = 1/6), which means that most of the C photoemission signal arises from carbon atoms belonging to either uncoated regions of the carbon fabric or covered by ultrathin, i.e. <~10-nm-thick, smooth PAni films looking featureless in SEM [36]. On the contrary, the N/C ratio is rather close to 1/6 at long electropolymerization time, indicating that XPS is mainly probing the surface chemistry of the polymeric fraction in the hybrid composite (Figure 4). Therefore, the evolution of the N/C ratio is in close agreement with SEM micrographs in Figure 2 and Figure 3a,b, which showed scattered compact PAni deposits at short electropolymerization time and thick PAni layers almost completely covering the carbon cloth substrate at long treatment time (120 min). At short to moderate electropolymerization time, the N/C values corresponding to analyzed areas on the fabric side exposed to the electrolyte were only slightly lower than those described above and changed in a similar fashion (open square circles in inset to Figure 4). This suggests that a smooth thin layer of PAni also forms on this face within this time interval, although it is indiscernible by SEM imaging under our experimental conditions. The long-term N/C atomic ratio (inset to Figure 4) is high but noticeably lower than at the electrode side, in line with the surface topography shown in Figure 3c,d. Finally, the S/C ratio parallels the N/C tendency (open circles in Figure 4), which strongly suggests that most of the sulfur present on the surface of the PAni-ACC composite plays a role as a dopant ion.

**Figure 5:**
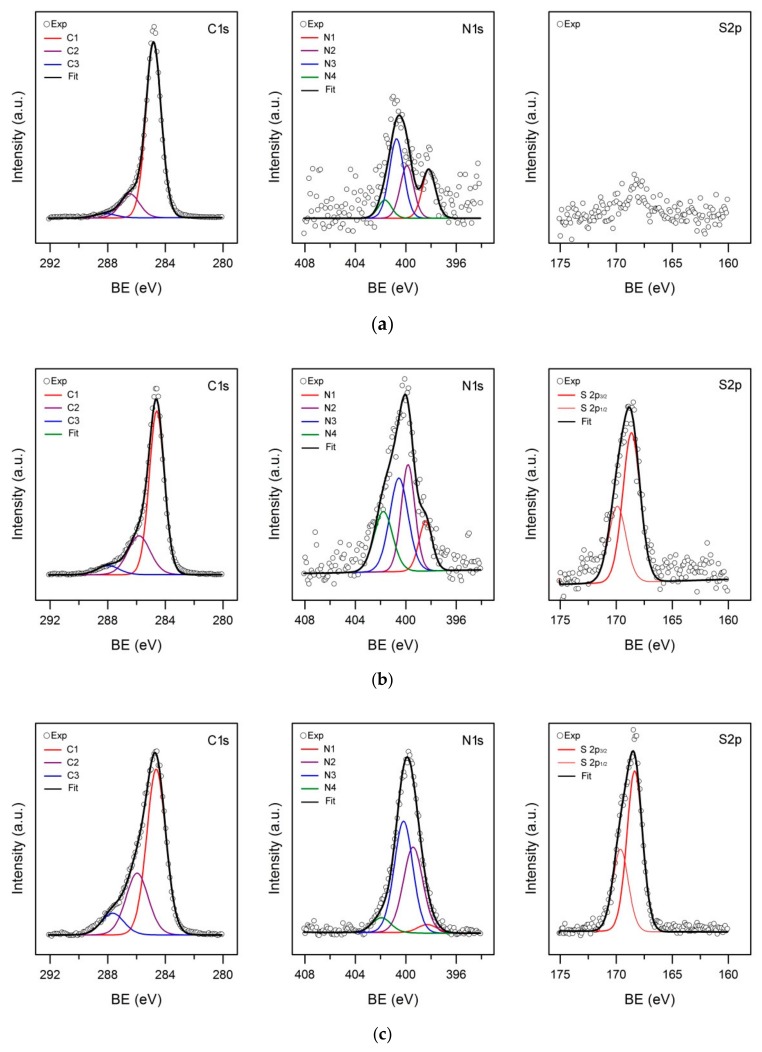
High-resolution C1s, N1s and S2p core-level X-ray photoelectron spectra of (**a**) untreated ACC and hybrid PAni-ACC composites synthesized after (**b**) 40 min and (**c**) 120 min electropolymerization time.

Figure 5 shows the high-resolution photoelectron spectra of C1s, N1s and S2p core levels of fresh (Figure 5a) and PAni-modified ACC after electropolymerization at different times (Figure 5b,c). The C1s peak was satisfactorily fitted with photoelectron contributions at average values of 284.6 ± 0.1 (C1), 285.9 ± 0.3 (C2) and 287.9 ± 0.2 eV (C3) respectively (Table S2). The C1 subpeak is the major component and corresponds to aromatic carbons in graphene layers of the carbon fabric [32,38,39,40] and also to those belonging to carbon rings in the polymer backbone [41]. The C2 subpeak can be assigned to carbon atoms singly bound to oxygen groups (i.e., C–OH/C–O–C functionalities) [38,40], but to some extent it can be contributed to by C–N and C=N/C=N^+^ groups from the polymeric fraction in the hybrid composite [41,42]. The chemical shift of the C3 subpeak is characteristic of double-bonded carbon–oxygen complexes (e.g., C=O functional groups) [38]. Also, C atoms singly bound to positively charged N atoms in PAni have been associated with this energy region [43].

In agreement with the low relative abundance of S, the S2p core-level spectrum of the fresh ACC sample (Figure 5a) shows a weak band centered at ca. 167.9 eV. Because of the low intensity of this band, fitting the possible components under the envelope was not attempted. According to the 10- to 30-fold increase in the amount of S (Table S1), S2p spectra in Figure 5b,c are much more intense. They appear shifted to higher BEs, therefore, pointing to a higher oxidation state of the S atom. The fitting of the S2p line revealed a single atomic environment with a spin-orbit doublet having its 2p_3/2_ component located at 168.6 ± 0.1 eV, so confirming the presence of (bi)sulfate anions [44].

The N1s photoemission line (Figure 5) can be decomposed into four distinct components located on average at 398.2 ± 0.1 (N1), 399.7 ± 0.2 (N2), 400.6 ± 0.2 (N3), and 402.0 ± 0.2 eV (N4), (Table S2). As far as activated carbon materials are concerned, the low-BE component was assigned to nitrogen in pyridine-like structures, and the N2 subpeak may be ascribed to aromatic amide or amine moieties, as well as to pyrrolic or pyridone structures [39,45]. The contributions above 400 eV correspond to positively-charged N structures: quaternary nitrogen (N3) and pyridine N-oxides (N4) [39,45]. By contrast, totally different local chemical states for N have been described in the literature dealing with N-containing conducting polymers, like polyaniline and its derivatives [5,41,42]: The N1 peak was attributed to deprotonated imine nitrogen in quinoneimine units, N2 to amine nitrogen in benzenoid rings, and peaks at >400 eV to positively charged N atoms. In particular, a peak component at about 400.5 eV (N3) was assigned to N atoms with delocalized positive charge, while that at the highest BE (N4) was associated with N atoms bearing localized positive charge. These assignments seem more appropriate to interpret the N1s core-level spectrum in Figure 5c, since the high N/C atom ratio suggests that XPS entirely reflects the composition of a layer of conducting polymer. The PAni doping level (S/N ratio) in the hybrid composite formed of Figure 5c is 0.63, and the protonation level (N^+^/N ratio) is 0.56. Therefore, sulfur is most likely to be incorporated in the form of (bi) sulfate anions, which compensates for the positive charge residing on nitrogen sites. Under the assumption that cationic N atoms originate solely from protonation of quinoneimine N sites [5], the proportion of neutral imine (=N−, 398.2 eV) plus charged nitrogen (N^+^, >400 eV) to total nitrogen gives an intrinsic redox state of 60%. The N1s line corresponding to PAni-ACC composites formed at intermediate electropolymerization time (Figure 5b) can also be resolved into the same four aforementioned components, but their true assignment may be obscured by N photoelectrons from the carbon matrix, which represents nearly 1/3 of the total N (Table S1). Therefore, an estimation of the doping level, protonation level and redox state of polyaniline was not attempted in this case.

*(2) Valence-Band Spectra*

**Figure 6:**
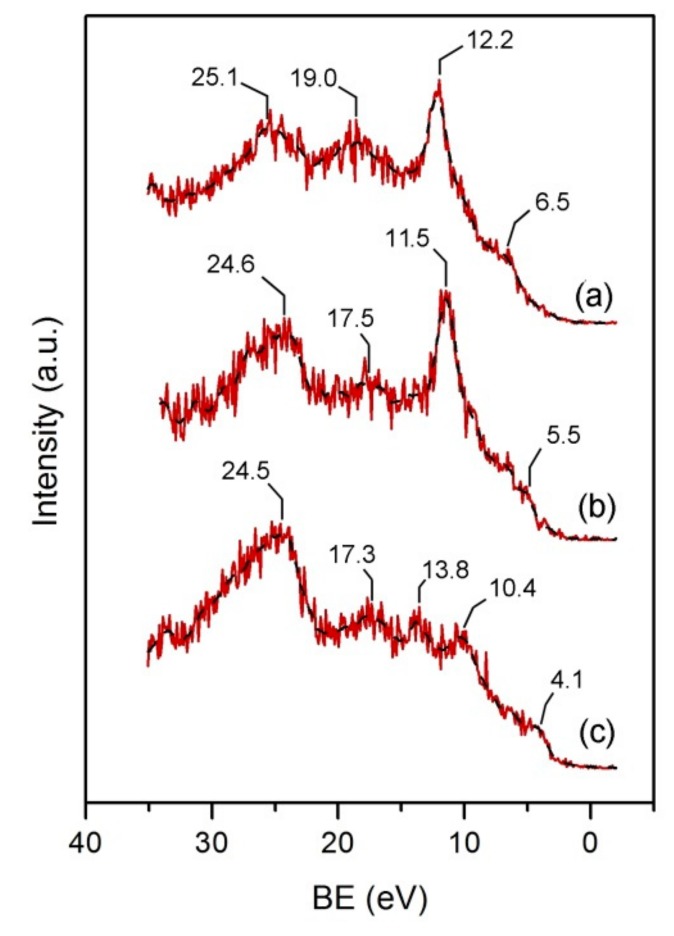
Valence-band X-ray photoelectron spectra of (**a**) untreated ACC and hybrid PAni-ACC composites synthesized after (**b**) 20 min and (**c**) 120 min of galvanostatic electropolymerization.

Figure 6 shows the variation in the Valence-band (VB) spectrum of the ACC substrate after modification with PAni. The spectrum of the bare carbon cloth (Figure 6a) is characterized by two broad peaks of similar relative intensity corresponding to O2s (25 eV) and C2s (19 eV) levels [40]. This latter peak may also involve a possible contribution from N2s orbitals at its low BE energy side (18–16 eV) [40]. The region below 16 eV is characterized by features resulting from the strong overlap of mixed O2p, C2p and N2p components [40]. In Figure 6a, a sharp peak appears in this region (12.2 eV), serving as a distinctive fingerprint for our base carbon fabric material. This characteristic peak is totally suppressed in the VB spectrum of hybrid PAni-ACC composites formed at prolonged electropolymerization time (Figure 6c, 120 min). The VB lineshape in this material shows three weak consecutive peaks laying in the region 10–20 eV and an asymmetric peak of high intensity at 24.5 eV. Moreover, it should be emphasized that the VB edge is far below the Fermi level in the ACC substrate, but the growth of PAni increases the density of states near the Fermi energy to delineate a small shoulder at ~4 eV. A similar VB pattern has been reported earlier for PAni films, although the relative peak intensity and resolution of the various peaks is highly dependent on the particular intrinsic redox state, the doping and protonation levels, and the nature of the dopant ion [46,47,48]. Nakajima et al. [46] reported a set of peaks in the region 10–20 eV (17.5 eV, 14 eV and 11.5 eV) due to an overlap of molecular orbitals involving benzenoid C and N in leucoemeraldine salt, while the electron density close to the Fermi level was found to be characteristic of a conjugated structure of double and single bonds. More recently, Bocchini et al. [48] distinguished a C2s peak at 13 eV, a 9–11 eV feature corresponding to a C–N 2p-σ state, and a group of weak photoemission peaks below 8 eV assigned to C2p-σ and C2p-π bands in polyemeraldine doped with camphorsulphonic or aminosulphonic acids. Hybrid PAni-ACC composites formed at intermediate electropolymerization times (Figure 6b) show mixed features from both the underlying carbon matrix and a polymer thin film. Thus, the lineshape, relative intensity and position of peaks above 15 eV change to become similar to that of PAni, whereas the characteristic sharp peak survives, but is shifted to lower BE. Also, the VB edge lies between that of the untreated ACC and that of the fabric coated with thick PAni layers.

#### 3.1.3. Porous Texture

**Figure 7:**
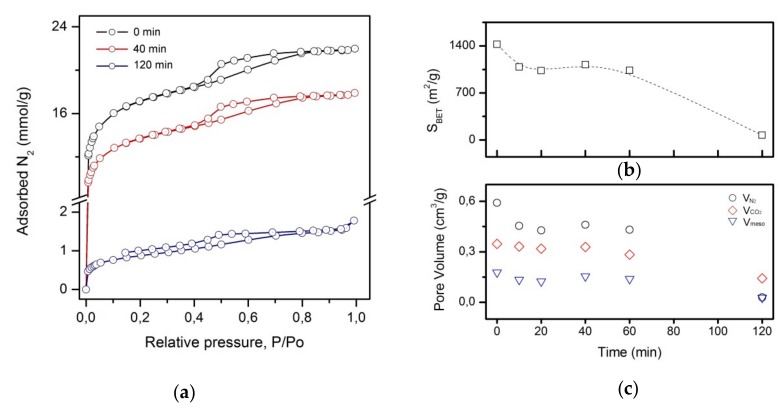
Porous texture characterization of untreated ACC and hybrid Pani-ACC composites synthesized after different electropolymerization times: (**a**) N_2_ adsorption–desorption isotherms; (**b**) Brunauer-Emmett-Teller (BET) surface area; (**c**) Pore volume distribution: micropore volume (open circles), ultramicropore volume (open diamonds), mesopore volume (open triangles).

Figure 7a shows nitrogen adsorption/desorption isotherms of bare ACC and representative PAni-ACC composite samples. The evolution of the BET specific surface area and that of different pore volumes with the electropolymerization time is plotted in Figure 7b,c respectively. A detailed list of these parameters can be found in Table 1. The shape of the N_2_ adsorption curve of the unmodified ACC (Figure 7a) corresponds to type IV isotherms, with a H4 adsorption/desorption hysteresis loop [49]. This form is typical of solids possessing mesopores embedded in a microporous matrix, where the adsorptive uptake proceeds via multilayer adsorption followed by capillary condensation [49]. The shape of the isotherm is similar to those reported for other cellulose-based commercial activated carbon fibers [23,39]. The growth of PAni layers leads to a general decrease in the N_2_ uptake, but the isotherm shape remains unchanged for composite textiles obtained at short to intermediate electropolymerization time. However, when the amount of charge passed is sufficiently high to produce thick PAni films, there is a dramatic loss in the N_2_ adsorption capacity and the hysteresis does not close at lower pressures. This may be due to diffusional restrictions to gas adsorption/desorption associated to pore narrowing and/or occlusion induced by the grown polymer.

The commercial ACC has a high apparent BET surface area of 1424 m^2^/g and exhibits both a micropore and mesopore structure (Table 1). In accordance to what is observed in Figure 7a, micro- and mesopore volumes, and therefore the specific surface area, decrease by 20%–25% whenever PAni grows as a uniform thin and dense film at short-to-moderate electropolymerization time (10–60 min, Figure 7b,c). Within this time interval, these parameters remain constant, but they drop pronouncedly in the hybrid composites with thick PAni layers. In contrast, the volume of the narrower micropores (ultramicropores) barely changes with PAni loading, except at long electropolymerization time, when a clear decrease is also observed (Figure 7c).

#### 3.1.4. Thermal Analysis

**Figure 8:**
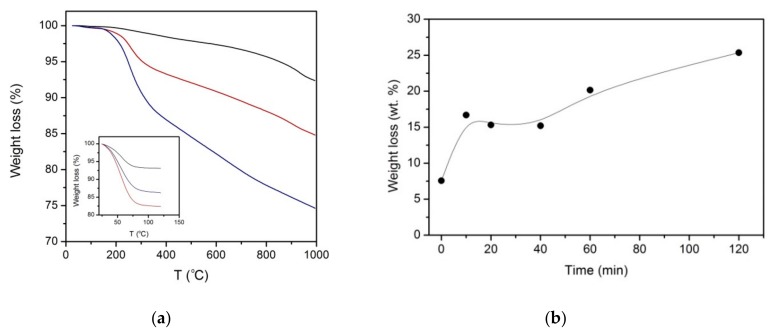
(**a**) TGA curves of unmodified ACC (black line) and hybrid PAni-ACC composites synthesized after 40 min (red line) and 120 min (blue line) of galvanostatic electropolymerization; the inset shows the thermal pattern during the first thermogravimetric run up to 120 °C; the main panel shows the thermal behavior of the resulting dried samples; (**b**) Evolution of the total weight loss at 1000 °C for heat-dried PAni-ACC composites obtained at different electropolymerization times.

The thermogravimetric curves of representative PAni-ACC composites obtained at selected electropolymerization times are shown in Figure 8a and compared to the thermal evolution of the untreated ACC. During the first thermogravimetric run (25–120 °C, inset to Figure 8a), the carbon support and the PAni-loaded samples all exhibit a weight loss below 100 °C. This loss is associated with the endothermic release of moisture or weakly adsorbed water solvent molecules, and perhaps with the evaporation of some residual monomers [50,51,52]. The amount of water lost by the carbon support is lower than the water released from any polymer-modified fabric, probably because PAni imparts a significant hydrophilic character. However, no clear pattern correlating the amount of deposited polymer and the water content was found. During the second thermogravimetric run from 25–1000 °C only negligible residual water is lost from the samples (Figure 8a).

The dry carbon cloth undergoes a first smooth mass loss in the range 200–400 °C, followed by a continuous decomposition and a more pronounced loss between 900–1000 °C (Figure 8a, black line). These features are well connected with the thermal decomposition of oxygen surface complexes in carbonaceous materials, which are known to evolve as CO_2_ and CO [30]. The thermogravimetric curves of hybrid PAni-ACC composites show a prominent loss between 150–350 °C and a subsequent steady weight decrease up to 1000 °C. The observed thermal pattern is a characteristic feature of PAni-like powders or films in their emeraldine salt (i.e., doped) state [51,52,53]. The sharp loss in the temperature range 150–350 °C has been attributed to either chain scission or cross-linking processes (e.g., leading to phenazine-like segments) with dopant removal [50,51,52], while the loss above 400 °C has been related to structural degradation of the polymer backbone leading to its complete carbonization at the highest temperature [52,53,54].

Our results show that both the sharp feature within 150–350 °C and the total weight loss are closely related to the amount of CP deposited on the carbon fabric (Figure 8a, red and blue lines). In samples with a moderate PAni loading (red line in Figure 8a), the loss corresponding to the decomposition processes of the underlying carbon cloth (800–1000 °C) is still discerned. However, it is missed in heavily-loaded PAni-ACC composites (Figure 8a, blue line). The total weight loss of dry samples as a function of the electropolymerization time (Figure 8b) reflects the evolution of the polymer amount in the hybrid material. Figure 8b shows an abrupt increase at the early stages of the electropolymerization process, a stabilization region and a second increase at the longest treatment time. Thus, the total weight loss vs. time plot mimics the evolution of the photoelectron N/C vs. time plot (Figure 4).

Thermogravimetric data can be used to estimate the PAni loading of the different hybrid composites. The estimate was done as follows: First, the total weight loss of dry hybrid PAni-ACC at 1000 °C was corrected by the loss corresponding to the carbon support at the same temperature (7.57 wt.%); then, the amount of polymeric material in the composite was estimated after considering that about 45 wt.% residue was left after carbonization at 1000 °C [53,54]. PAni-loadings are expressed either as the mass of polymer (mg) per unit geometric area (cm^2^) of carbon fabric or as wt.%. The results are summarized in Table 2 for composites electrosynthesized at 10–120 min time. The data confirm that a massive deposit of PAni occurs at 120 min of electropolymerization that accounts for ca. 50% of the composite material mass.

### 3.2. Enhanced Electrical and Adsorptive Properties.

#### 3.2.1. Capacitance and Surface Sheet Resistance.

**Figure 9:**
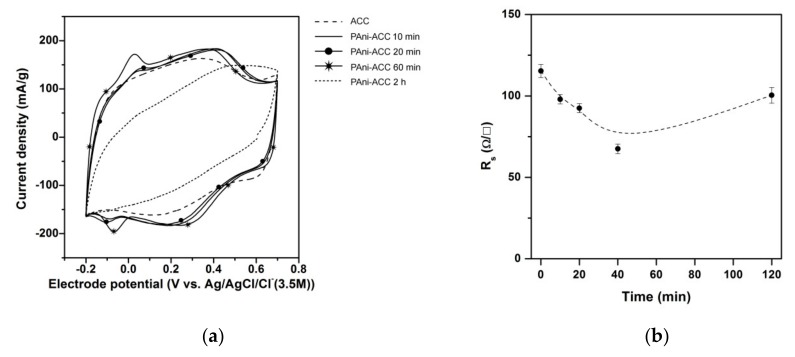
(**a**) Stabilized cyclic voltammograms of untreated ACC and hybrid PANi-ACC composites synthesized at different electropolymerization times. Scan rate: 1 mV·s^−1^, supporting electrolyte 1.0 M H_2_SO_4_; (**b**) Evolution of the surface sheet resistance upon the time of electropolymerization.

The electrical properties of the untreated ACC and PAni-coated hybrid composites were obtained from CV and four strip probe conductivity measurements (Figure 9). CVs became stabilized within two cycles and remained unchanged thereafter. No loss of material was seen during CV recording.

The CV of the untreated ACC (Figure 9a), dashed line) shows a capacitive response with a distorted rectangular shape that indicates a deviation from the ideal double-layer capacitor behavior [11,15]. This is a symptom of a slow charging/discharging response caused by a potential difference across micropores [18,55]. In addition to the main double layer charge/discharge contribution, a redox couple is discerned (E_1/2_ = 0.25 V). This feature is associated with a faradic process involving redox transitions of surface carbon–oxygen groups, thus behaving as a pseudocapacitance [11,30]. At low or intermediate electropolymerization time (0–60 min), a moderate increase in the voltammetric current occurs as a result of PAni film formation. This increase in current has a pseudocapacitive character and is mainly due to reversible redox transitions corresponding to the doping/dedoping processes in PAni. The broad redox couple located within 0.4–0.2 V has been assigned to the reversible transition between leucoemeraldine and emeraldine salt oxidation states of the polymer in several PAni-coated 3D porous carbon materials, like foams [15] and activated carbon fibers or clothes [11,22,55]. The pair of sharp and small peaks at ~0.0 V may be related to the presence of some phenazine- or phenoxazine-like moieties in the PAni backbone [41,56,57]. In accordance with the modest increase in the CV response, the specific capacitance of the PAni-ACC composites rises by up to a 12% (Table 3). Most of this increase occurs within the first 10 min of electropolymerization and then it tends to level off during the first hour of electropolymerization. The CV corresponding to thick PAni coatings formed at the longest treatment (Figure 9a, dotted line) appears noticeably tilted. Furthermore, the pseudocapacitive features of PAni are barely distinguishable and the total specific capacitance falls even below the capacitance of the unmodified ACC (Table 3). The abrupt decrease in the specific capacitance parallels the loss of available BET surface area measured by N_2_ adsorption isotherm. The evolution of surface sheet resistance with time (Figure 9b) follows up the reported changes in the CV response and the specific capacitance. Within the first 60 min of electropolymerization, the electrical resistance of the hybrid PAni-ACC composite diminishes, but it rises again at prolonged treatment, i.e., when a thick nanofibrous PAni coating develops.

#### 3.2.2. Adsorption of Acid Red 27

**Figure 10:**
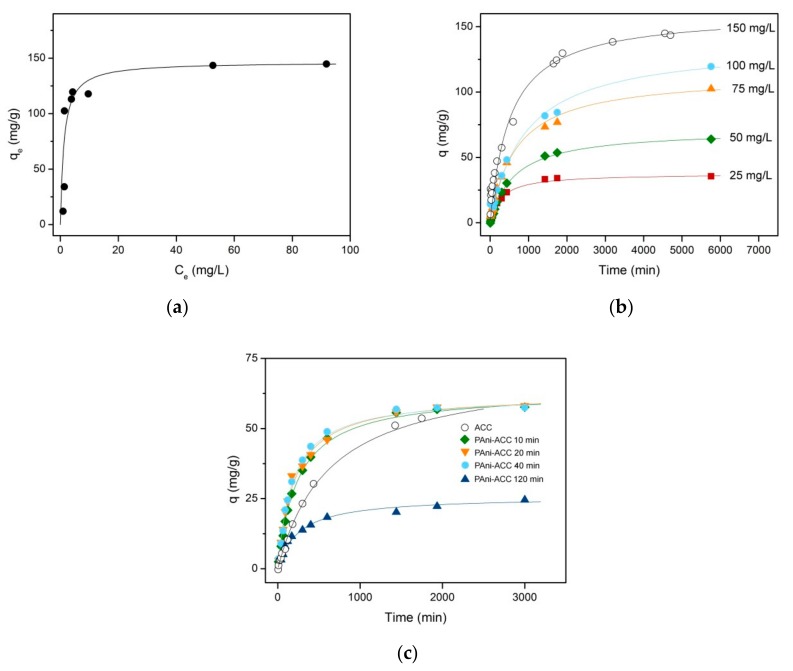
(**a**) Experimental (solid symbols) and Langmuir-based simulated adsorption isotherm (solid line) for Acid Red 27 on untreated ACC at 25 °C; (**b**) Experimental (symbols) and PSO modelled (solid lines) kinetic curves for the adsorption of Acid Red 27 on untreated ACC at different initial dye concentrations; (**c**) Experimental (symbols) and PSO modelled (solid lines) kinetic adsorption curves of 50 mg L^−1^ Acid Red 27 solution onto hybrid PAni-ACC composites formed at different electropolymerization times.

The experimental equilibrium data for the uptake of Acid Red 27 on unmodified ACC (Figure 10a) at 25 °C were fitted with the Langmuir and Freundlich models (see Appendix A), [58,59]. The isotherm parameters can be obtained from the slope and y-intercept of the *C_e_/q_e_* vs. *C_e_* and ln *q_e_* vs. ln *C_e_* plots. The correlation coefficients for the linear regression fittings of Langmuir and Freundlich models to the experimental data were 0.9997 and 0.9397, respectively (Figure S1). Therefore, the equilibrium adsorption of Acid Red 27 on ACC is best described by the Langmuir isotherm, with a maximum adsorption capacity of 146.6 mg g^−1^ and *b*=0.82 L mg^−1^. The simulated adsorption equilibrium curve based on Langmuir parameters is also shown in Figure 10a (solid line).

The isothermal kinetic curves for the adsorption of Acid Red 27 on ACC are shown in Figure 10b for concentrations ranging 25–150 mg L^−1^. The dye uptake is faster at the initial stages of adsorption and then it decreases as the process approaches equilibrium, owing to the increasing occupancy of surface sites and slow diffusion into the internal porous structure [14]. The adsorption rate increases with the increasing initial concentration. The equilibrium uptake shows the same dependence, although it levels off at high initial concentrations because maximum adsorption capacity is approached.

Kinetic data in Figure 10b were modelled according to the well-known pseudo-first order (PFO or Lagergren equation) and Ho’s pseudo-second order (PSO) models (Equations (A5) and (A6), Appendix A) [60]. The relevant kinetic parameters can be inferred from the slope and y-intercept of ln(*q_e_* − *q*) vs. *t* (PFO model) and *t/q* vs. *t* (PSO model) plots (see Figures S2 and S3, respectively). The calculated rate constants, the theoretically predicted *q_e_*, and the corresponding *R*^2^ are listed in Table 4 for different initial Acid Red 27 concentrations. Also tabulated are the calculated PSO initial adsorption rates, *r*_0_. Both models provide a good fitting of the experimental data over the whole time span according to the square correlation coefficient. Then, the goodness of fit was further evaluated with the so-called average relative error (ARE, Equation (A10), Appendix A). The ARE in Table 4 is lower for PSO-based fittings than for PFO kinetics over the whole range of initial concentrations. Further, the equilibrium uptakes predicted by the PSO model are generally closer to the experimental ones. Then, the PSO model correlates the experimental kinetic data better than the PFO model does. The second order rate constant shows a decreasing tendency as the initial concentration increases, whereas the initial adsorption rate follows a growing trend. The simulated adsorption curves derived from PSO kinetics are drawn in Figure 10b as solid lines.

The PSO model is known to show widespread good fit to the adsorption kinetics of most dyes and organic pollutants on activated carbon and other synthetic or natural adsorbents [9,60]. However, it has been claimed [60] that the rate constant, *k*_2_, should not be regarded as being truly representative of intrinsic kinetics, but as an empirical parameter that lumps different controlling mechanisms in a complex manner. In fact, our data invariably show a noticeable deviation from linearity at the initial stage of adsorption (Figure S3). This typical downward curvature was previously realized by Azizian et al. [61], who ascribed it to a mixed rate control by both diffusion and surface reaction. In order to check the significance of mass transfer on the Acid Red 27 adsorption kinetics onto ACC, we used the Vermeulen model (Equation (A9), Appendix A) [60]. According to this model, the *Bt* vs. *t* plot (Boyd plot) should be a straight line passing through the origin for a pure intraparticle diffusion mechanism. If the line has a non-zero intercept, the adsorption is also controlled by external film diffusion [31,60]. In our case, the plots are linear for the whole concentration range (see Figure S4), but they do not pass through the origin (Table S3), thus pointing out that film diffusion is also involved in governing the adsorption rate.

The evolution of Acid Red 27 adsorption uptake onto different PAni-ACC hybrid composites is shown in Figure 10c for a single dye initial concentration of 50 mg L^−1^. The kinetic curve for the bare ACC is also depicted for the sake of comparison. PAni layers deposited at short time (thin and compact films) remarkably enhance the rate of adsorption, but the total amount of dye adsorbed at equilibrium remains basically unchanged. However, higher PAni loading achieved at prolonged electropolymerization (thick and porous nanofibrous coating) leads to both a decrease in the adsorption rate and a considerable loss of the adsorption capacity at equilibrium. As above, PFO and PSO modelling of the experimental data were attempted and the kinetic parameters of interest, as well as proofs for testing the goodness of fit, are summarized in Table 5. Again, the PSO model provides an overall better description of the adsorption kinetics. Solid lines in Figure 10c were then traced by using the calculated kinetic parameters of the PSO fitting. These parameters corroborate that hybrid PAni-ACC composites of low polymer loading and smooth film morphology promote a faster adsorption of the dye, while the equilibrium uptake remains unaffected. On the contrary, the nanofibrous thick PAni coating is detrimental to both the adsorption rate and the dye loading into the adsorbent composite material.

Finally, the involvement of mass transfer on the adsorptive capability of the hybrid composites was evaluated with the aid of *Bt* vs. *t* plots (Figure S5 and Table S4). In this instance, only the composites formed at 10 to 60 min of electropolymerization show good linear plots with a non-zero intercept, which again suggests that both external and intraparticle diffusion may play a role in the adsorption process. Instead, the materials obtained at longer treatment time do not show a linear behavior and therefore the involvement of mass transfer is unclear.

## 4. Discussion

In this work, we show that large sized (4 cm × 5 cm) activated carbon cloths can be easily modified with electrochemically deposited polyaniline by closely attaching the fabric to a stainless steel anode in a filter-press type cell. A simple galvanostatic procedure at a constant current of 100 mA (~14 mA g^−1^ cm^−2^) allowed the electropolymerization process to be carried out at an anodic potential varying in the range 0.6–0.8 V vs. Ag/AgCl. This electrode potential is sufficient to oxidize aniline monomers, to generate enough PAni nucleation sites and to ensure fast chain growth [16,55,62]. A simple control of the PAni loading could be achieved by passing different amounts of charge, i.e., by adjusting the elapsed electropolymerization time. The highly porous carbon fabric provides the hybrid composite material with high specific surface area, flexibility and mechanical strength to mitigate the stress associated with polymer volume changes accompanying doping/dedoping [11,12,22], while PAni adds some new enhanced characteristics related to its reversible redox properties and electron conductivity [2,12,62].

It is well known that PAni and its composites can be synthesized with a wide variety of micro- and nanoscale structures, with a surprising number of different sizes and shapes, from smooth thin films to nanofibers, nanorods or nanotubes to globular or granular agglomerates [26,27]. The observed supramolecular organization and structure is strongly dependent on the method of synthesis and the experimental conditions [6,26,27]. As for electrochemical methods, the applied current/potential and the way the electrode is polarized (i.e., potentiostatically, galvanostatically, by cyclic potential sweep or pulses), as well as the nature and surface condition of the electrode support, all have a dramatic effect on the morphology of the deposited polymer film [6]. In the present work, the film morphology varied with processing time from dense and uniform thin films covering individual carbon fibers to a loosely adherent and porous, thick coating of nanofibrous or nanowire-shaped structures. Three-dimensional networks of nanofibrilar or nanowire structures seem the most frequently encountered morphology for PAni deposited on high surface area carbon substrates (e.g., carbon fibers or cloths), either by electrochemical [20,21,62,63] or conventional in-situ chemical polymerization [22,23,24]. However, in agreement with our results, some authors have obtained smooth dense layers [8,63] or a transition from this morphology to irregular or randomly connected nanofibrilar aggregates upon extending the polymerization time [12,62]. It has also been reported that long reaction time [23] or prolonged galvanostatic oxidation [20] lead to incontrollable growth among carbon fibers and result in a detrimental effect via peeling-off from the carbon surface.

Apart from the scattered compact deposits seen in Figure 2, no clear evidence of a PAni film uniformly covering the carbon fibers is provided by SEM imaging. However, the increase in the surface N/C ratio measured by XPS at both sides of the carbon cloth suggests that an (ultra)thin film develops within this timespan. Also, the PAni loadings deduced from thermogravimetric analysis (Table 2) indicate that there is more polymer than what is seen by SEM. We believe that during the pre-conditioning step, anilinium cations are pre-adsorbed on the internal porosity of the carbon cloth [11,55], thus acting as nucleation seeds and reacting with liquid-phase aniline to yield ultra-thin films covering the pore walls. This view agrees with the results by Salinas-Torres and coworkers [17], who used position-resolved micro-Small Angle X-Ray Scattering (SAXS) to demonstrate that a PAni layer of sub-nanometric thickness grows inside the microporosity of activated carbon fibers. The particular non-linear increase in the PAni content revealed by both XPS and thermal analysis (Figure 4 and Figure 8, Table 2) might be explained by a decrease in the polymerization rate caused by the depletion of aniline monomers inside the pores and the induced concentration-gradient between the external bulk solution and the solution filling the pores. Note that dye adsorption studies point out that the bare carbon material possesses a large pore mass transfer resistance (see below). Slow internal pore diffusion provides enough time for self-assembly of intermediate oligomers into thin films. Once PAni reaches the outer carbon surface, external film diffusion facilitates fast access of solution aniline to the growing chains and the polymerization rate increases again to build a 3D network of nanofibrilar structures and nanowire aggregates.

As long as a thin film develops on the carbon fabric surface, XPS shows mixed spectroscopic characteristics from both the support and PAni. When a thick coating is formed, XPS is typical of pure PAni, close to the semioxidized emeraldinde salt state. The temperature-induced changes in the hybrid composites (Figure 8) are also compatible with the thermal behavior of PAni in its doped emeraldine salt state [50,51,52,53]. The evolution with the electropolymerization time of the dry composite total weight loss and the calculated PAni loading (Figure 8 and Table 2) are consistent with the trends shown by XPS analysis. The PAni loadings achieved in our hybrid composites ranged from ~5 (within the first 40 min of treatment) to 18 mg cm^−2^, at the longest polymerization time. These loadings are in the range of those reported by others for carbon fiber substrates [11,23,62,64].

The electrodeposition of PAni is accompanied by a decrease in the apparent BET area and pore volume. Earlier authors also described a remarkable blockage of micro- and mesoporosity in composites of activated carbon fibers [8,11,17] or activated carbon fiber textiles [23,25,64] with PAni films, deposited either by chemical or electrochemical methods. In these reports, the surface area loss ranged from 20% to 40%. In the present investigation, the specific surface area decreases by ca. 20%–25% and remains basically unchanged within the time interval when a uniform and dense thin PAni film covers the carbon surface. The evolution of pore volumes (Table 1 and Figure 7c) indicates that PAni does not significantly grow inside the narrower micropores, in contrast to the results by Salinas-Torres and coworkers [17]. There is no clear evidence why polymerization inside ultramicropores is hampered, but it may arise from too short a contact time in the pre-conditioning step to allow diffusion of aniline into such narrow pores. Also, we can tentatively argue that carbon fibers may possess some kind of hierarchical pore structure, with ultramicropores preferentially distributed near the fiber core. Thus, PAni films blocking the outer micro- and mesopores would hinder efficient monomer supply to sustain polymerization inside the inner pores. In fact, position-resolved micro-SAXS studies revealed a heterogeneous radial distribution of electrosynthesized PAni [17], which preferentially accumulates in the outer shells of carbon fibers. The thick nanofibrous PAni coating grown at prolonged electropolymerization time virtually blocks all the porosity, thereby causing an abrupt decrease in the specific surface area to reach a typical value of pure PAni precipitates [65].

The reversible redox transitions of PAni doping/dedoping processes contribute to moderately enhance the capacitive response of hybrid PAni-ACC composites. The extra pseudocapacitive features observed in the CVs of the hybrid composites (Figure 9) arise from the leucoemeraldine-to-emeraldine state transition (0.2–0.4 V) and a reversible electron-transfer (~0.0 V) probably involving phenazine- or phenoxazine-like segments in the polymer backbone. These latter redox features appear in polymeric materials of the PAni family, like oligomers of o-aminodiphenylamine [41,56], ladder polymers like poly-o-phenylenediamine [57] or poly-o-aminophenol [56], PAni- and poly-o-anisidine-clay nanocomposites [66,67] or reduced graphene oxide-PAni-ACC composites [63]. More recently, it has been claimed that even chemically synthesized PAni contain constitutional phenazine and N-phenylphenazine segments [68] that play a key role in the self-assembly of polymer chains to build different supramolecular structures [27].

The specific capacitance of the hybrid composites increases moderately in hybrid composites with thin dense PAni films. (Table 3). The reported specific capacitance for pure Pani-modified electrodes lies within a broad range from 160 to 815 F/g [69], while the theoretical capacitance for Pani at a 50% doping level is 750 F/g [70]. Upon considering this latter value, the expected capacitance for a 20 wt.% PAni-ACC composite would be about 250 F/g, but if one considers the lowest reported PAni capacitance, an estimated value of about 134 F/g would be obtained, which is close to the capacitances listed in Table 3. Then, the slight increase in composite capacitance could be due to the electrodeposition of PAni with a small specific capacitance. Alternatively, it can also be a consequence of the deleterious effect of decreasing the available surface area, which counteracts both the PAni extra capacitance and the reduced sheet resistance. Composites with the highest PAni loading densities show a strongly distorted CV and a decreased capacitance, as a consequence of the increased sheet resistance and the abrupt diminution of the surface area caused by the thick nanofibrous coating. Note that VB analysis revealed that the nanofibrous PAni layers have a higher DOS near the Fermi level (Figure 6). Then, the increased *R*_s_ and lessened electrochemical properties imparted by nanofibrous PAni layers should not be caused by poor intrinsic electron conductivity of PAni, but related to high PAni–PAni and/or PAni-carbon intraparticle resistance to charge transport.

The evolution of the specific capacitance observed in this work is in agreement with the results reported earlier by other researchers [12,16,21,23,62]. It has been generally shown that the capacitance is enhanced by either smooth homogeneous PAni coatings of small thickness [12] or nanofiber-shaped assemblies with a certain degree of 3D order and small diameter [22,23] obtained at low PAni loading densities. Such thin nanostructures are believed to provide fast access of electrolyte and shortened path lengths for ion and electron transport. When the loading is raised at longer polymerization times and/or higher monomer concentration, thicker polymer films build up with a high degree of particle agglomeration [12,23,24]. This results in an increase of the diffusion resistance of electrolyte ions and also in a less efficient electrical contact between the conducting polymer and the textile carbon [22].

Finally, we studied the effect of the modification of the ACC by PAni on the adsorption of Acid Red 27. The dye uptake on both bare ACC and PAni-ACC composites obeys PSO kinetics, with the involvement of mass transport on the adsorption rate control. Our results point out that PAni thin films of moderate loading densities provide accelerated PSO adsorption kinetics of Acid Red 27 from aqueous solution, while keeping the maximum adsorption capacity unchanged. However, the values of *k*_2_ (Table 5) and the apparent intraparticle diffusion coefficients from Boyd plots (Table S4) are still below the range of those commonly reported for dye adsorption on many different adsorbents (in the order of 10^−2^–10^−4^ g mg^−1^min^−1^ and ~10^−17^ m^2^ s^−1^, respectively) [14,65,71,72,73]. These low values signify that the internal mass transfer resistance is very high and is dominated by the carbon porous structure, thereby explaining the very long time taken by the adsorption system to attain equilibrium. The adsorption of dyes on PAni powder [10,74] or its nanocomposites with other conducting polymers [65], carbonaceous materials, metal oxides, and low-cost bioadsorbents [9,14,19] has been extensively studied to date, with a general consensus that the adsorption rate is governed by PSO kinetics. The uptake of dyes on PAni-based adsorbents seems to be driven by different types of binding forces, namely, π–π attraction, hydrogen bonding or electrostatic interaction between charges residing on both the PAni backbone and the dye functional groups [9,14]. In our case, the electrostatic interaction between negatively-charged sulfonate groups in Acid Red 27 and positively-charged N sites in the backbone of the acid-doped PAni films overrides the loss in the specific surface and leads to adsorption improvement. Further, the exchange of some (bi) sulfate ions with dye molecules as dopant anions should not be ruled out as an additional mechanism to promote their incorporation to the hybrid adsorbent. A similar explanation was put forward by other authors for the promoted adsorption of anionic sulfonated dyes on PAni [9,10] or PAni-based nanocomposites [19,65]. Despite the fact that the same interaction forces are operative when a thick nanofibrous network of PAni is formed at the longest treatment time, the strong blockage of carbon pores and the abrupt decrease in the specific surface area resulted in an important adsorption rate slowdown and a dramatic loss in the maximum adsorption capacity.

## 5. Conclusions

Flexible PAni-ACC composites of relatively large size can be easily fabricated by simple galvanostatic electropolymerization of dissolved aniline in a filter-press electrochemical cell, whose modular and stackable design is particularly well suited for easy scaling-up to pilot plant or even technical scales. The morphology of the deposited PAni is strongly dependent on the polymerization time (i.e., the amount of charge passed), ranging from dense and thin films to porous and thick coatings made of interconnected nanofibrils or nanowires. With the exception of some scattered spots, thin films appear featureless in SEM, and their occurrence is indirectly deduced from the XPS N/C and S/C ratios. Composites with a polymer thin film morphology show mixed XPS and valence band patterns from the carbon fabric and the conducting polymer, whereas those for composites with PAni nanofibrous morphology are typical of pure PAni, in a state close to that of (bi) sulfate-doped emeraldine salt.

PAni loadings range from 5 to 18 mg cm^−2^ (~25–50 wt.%), but they increase non-linearly with the electropolymerization time, as shown by combined XPS and TGA data. We propose that aniline polymerization occurs initially inside the carbon pores, but the polymerization rate levels off once aniline is depleted and supplied by slow internal pore diffusion. This mechanism would also facilitate the self-assembly of aniline oligomers into thin films. The analysis of the N_2_ and CO_2_ isotherm data indicates that thin film PAni deposition occurs on micro- and mesopores, while the ultramicropore volume remains unaffected. Accordingly, the BET surface area decreases by 20%–25%. Thick nanofibrous coatings formed in highly-loaded PAni-ACC composites cause a strong blockage of pore entrance and a dramatic loss of the specific surface area to values typical of pure PAni.

The conductivity, electrochemical and liquid-phase adsorptive properties of the hybrid PAni-ACC composites are strongly conditioned by the microstructure of deposited PAni. The cyclic voltammograms of composites with thin film morphology show pseudocapacitive features related to reversible leucoemeraldine-emeraldine transition and electron-transfer in phenazine/phenoxazine-like segments. Accordingly, the specific capacitance is enhanced and the sheet resistance falls to a minimum. In addition, the pseudo-second order kinetics for the adsorption of Acid Red 27 are remarkably promoted. We believe that the extra pseudocapacitance, the improved conductivity and the attractive electrostatic interactions between dye molecules and PAni counterbalance the loss in a specific surface area. On the contrary, in composites with a thick nanofibrous morphology, the pronounced decrease in surface area, and perhaps higher PAni-PAni intraparticle and/or PAni–carbon contact resistances, can be at the origin of the decreased capacitance, conductivity, and adsorption rate and capacity.

## Figures and Tables

**Table 1:** Porous texture analysis of as-received ACC and hybrid PAni-ACC composites synthesized after different electropolymerization times.

Time (min)	S_BET_ (m^2^/g)	V_tp_ (cm^3^/g)	V_μ_^1^ (cm^3^/g)	V_ultra μ_^2^ (cm^3^/g)	V_meso_ (cm^3^/g)
0	1424	0.77	0.59	0.35	0.18
10	1086	0.59	0.45	0.33	0.14
20	1032	0.55	0.43	0.32	0.12
40	1120	0.61	0.46	0.33	0.15
60	1037	0.57	0.43	0.28	0.14
120	71.1	0.055	0.028	0.14	0.027

^1^ Volume of micropores. ^2^ Volume of ultramicropores.

**Table 2:** PAni loadings of dried hybrid PAni-ACC composites synthesized after different electropolymerization times.

Time (min)	Initial Dry Mass (mg)	Corrected Weight Loss ^1^ (%)	PAni Weight Loss (mg)	PAni Total Mass ^2^ (mg)	PAni Loading	
					(wt.%)	(mg cm^−2^) ^3^
10	8.8250	9.12	0.8047	2.2679	25.70	6.23
20	9.2770	7.75	0.7186	2.0251	21.83	5.03
40	9.9108	7.63	0.7561	2.1307	21.50	4.93
60	8.4070	12.58	1.0577	2.9807	35.46	9.89
120	11.1129	17.79	1.9766	5.5704	50.13	18.1

^1^ After subtraction of the total loss from the carbon fabric support (see text). ^2^ After considering that a 45 wt.% polymer residue remains on the heat-treated sample. ^3^ Calculated on the basis of cloth aerial density.

**Table 3:** Gravimetric specific capacitance of hybrid PAni-ACC composites as a function of the electropolymerization time.

Time (min)	C_sp_ (Fg^−1^)
0	127
10	138
20	137
40	136
60	142
120	98

**Table 4:** Pseudo-first-order and pseudo-second-order kinetic parameters for the adsorption of Acid Red 27 on ACC (*C*_0_: mg·L^−1^; *q_e_*: mg·g^−1^; *k*_1_: min^−1^; *k*_2_: g·mg^−1^·min^−1^; *r*_0_: mg·g^−1^·min^−1^).

C_0_	q_e,exp_	PFO				PSO				
		*k*_1_ × 10^3^	*q_e,cal_*	*R* ^2^	ARE	*k*_2_ × 10^5^	*q_e,cal_*	*r*_0_	*R* ^2^	ARE
25	35.5	1.85	32.4	0.993	0.35	9.21	37.6	0.130	0.997	0.16
50	63.9	1.05	60.5	0.991	0.27	2.24	71.2	0.113	0.997	0.07
75	102.5	0.79	94.0	0.976	0.37	1.80	109.1	0.215	0.982	0.22
100	119.6	0.67	105.8	0.982	0.69	0.94	134.0	0.169	0.987	0.37
150	143.5	1.05	122.7	0.994	0.52	1.94	153.1	0.454	0.995	0.21
200	144.7	1.14	137.2	0.994	0.26	1.10	163.6	0.294	0.999	0.13

**Table 5:** Pseudo-first-order and pseudo-second-order kinetic parameters for the adsorption of Acid Red 27 (50 mg L^−1^) on hybrid PAni-ACC composites synthesized at different electropolymerization times. (*t*: min; *q_e_*: mg·g^−1^; .*k*_1_: min^−1^; *k*_2_: g·mg^−1^·min^−1^; *r*_0_: mg·g^−1^·min^−1^).

t	q_e,exp_	PFO				PSO				
		*k*_1_ × 10^3^	*q_e,cal_*	*R* ^2^	ARE	*k*_2_ × 10^5^	*q_e,cal_*	*r*_0_	*R* ^2^	ARE
0	63.9	1.05	60.5	0.991	0.27	2.24	71.2	0.113	0.997	0.07
10	57.6	2.20	48.3	0.994	0.17	6.53	63.1	0.260	0.999	0.05
20	58.1	2.29	47.3	0.990	0.18	7.96	62.6	0.312	0.999	0.06
40	57.5	3.29	52.7	0.996	0.13	8.89	61.9	0.340	0.999	0.07
120	24.5	1.05	16.8	0.919	0.23	1.53	25.8	0.102	0.995	0.08

## Supplementary Materials

The following are available online at https://www.mdpi.com/1996-1944/12/16/2516/s1, Figure S1: Isotherm linearized fittings for Acid Red 27 adsorbed on ACC, Figure S2: Pseudo-first order ln(*q*_e_ − *q*) vs. *t* plots for the adsorption of Acid Red 27 on ACC, Figure S3: Pseudo-second order *t/q* vs. *t* plots for the adsorption of Acid Red 27 on ACC., Figure S4: Boyd plots for the adsorption of Acid Red 27 on ACC, Figure S5: Boyd plots for the adsorption of Acid Red 27 on hybrid PAni-ACC composites, Table S1: Elemental surface composition (in at.%) of ACC and hybrid PAni-ACC composites, Table S2: C1s and N1s peak positions and relative abundance, Table S3: Boyd plot parameters for the adsorption of Acid Red 27 on ACC, Table S4: Boyd plot parameters for the adsorption of Acid Red 27 on hybrid PAni-ACC composites.

## Appendix A

### Appendix A1. Specific Capacitance, C_sp_

The gravimetric specific capacitance was obtained from CV according to Equation (A1)

(A1) $$C_{sp}=\frac{\int \left(I/m\right)dE}{\nu \;∆E}$$

where *I/m* (mA g^−1^) is the voltammetric current normalized by the total mass of the composite material, ν is the scan rate (mV s^−1^), and Δ*E* (mV) is the potential window spanned in the CV. The reported capacitances are the average of capacitances calculated in both the anodic and cathodic branches of the voltammogram.

### Appendix A2. Surface Sheet Resistance, R_s_

To measure *R_s_*, the electrical resistance, *R* (Ω), was first obtained from the linear slope of *V* vs. *I* plots in the range 0–10 mA. Then, *R_s_* was calculated by using Equation (A2):

(A2) $$R=\frac{\rho }{t}\frac{L}{W}=R_{s}\frac{L}{W}$$

where *ρ* is the resistivity, *t* is the thickness of the probed carbon composite sheet, and *L* and *W* stand for its length and width, respectively.

### Appendix A3. Adsorption Isotherms and Kinetic Models

Langmuir (Equation (A3)) and Freundlich (Equation (A4)) isotherm models were used in their linearized forms:

(A3) $$\frac{C_{e}}{q_{e}}=\frac{1}{bq_{m}}+\frac{C_{e}}{q_{m}}$$

(A4) $$\ln q_{e}=\ln K_{F}+\frac{1}{n}\ln C_{e}$$

where *q_e_* (mg g^−1^) and *C_e_* (mg L^−1^) are the adsorbate uptake and solution concentration at equilibrium; and *q_m_* (mg g^−1^) and *b* (L mg^−1^) are the Langmuir parameters, representing the maximum adsorption capacity and the adsorption equilibrium constant respectively [59]. In the Freundlich model, the adsorption coefficient, *K_F_* (mg^(1 − 1/n)^L^1/n^g^−1^), is related to the adsorption strength, and the exponent *n* accounts for the energetic heterogeneity of the adsorbent surface [59].

PFO (Equation (A5)) and PSO (Equation (A6)) kinetic models were used in their respective linear forms:

(A5) $$\ln\left(q_{e}-q\right)=\ln q_{e}-k_{1}t$$

(A6) $$\frac{t}{q}=\frac{1}{k_{2}q_{e}^{2}}+\frac{t}{q_{e}}$$

where *q* and *q_e_* (mg g^−1^) are the adsorption uptakes at time *t* (min) and at equilibrium respectively, and *k*_1_ (min^−1^) and *k*_2_ (g mg^−1^ min^−1^) stand for the pseudo-first and pseudo-second order rate constants. In the PSO model, the product $k_{2}q_{e}^{2}$ is the initial adsorption rate, *r*_0_.

The Vermeulen model is an approximate solution of the exact Crank equation for intraparticle diffusion:

(A7) $$F\left(t\right)=\sqrt{1-\exp\left(-Bt\right)}$$

where *F(t)* is the fractional uptake (*q/q_e_*) at time *t*, and *B* is the time constant (min^−1^) that depends on the adsorbent particle radius, *r*_p_, and the effective intraparticle diffusion coefficient, *D*_e_, as follows [72,73]:

(A8) $$B=\frac{D_{e}\pi^{2}}{r_{p}^{2}}$$

The Vermeulen model is valid over a wide adsorption timespan and is commonly applied in the rearranged form:

(A9) $$Bt=-\ln\left(1-F^{2}\right)$$

Then, the product *Bt* is calculated from *F* at each contact time, and next plotted against time.

### Appendix A4. Average Relative Error

The average relative error (ARE) is defined as [60]:

(A10) $$ARE=\frac{1}{N}\overset{N}{\underset{i=1}{\sum }}\left|\frac{q_{i,exp}-q_{i,cal}}{q_{i,exp}}\right|$$

where the subscripts *exp* and *cal* refer to the experimental and calculated values, and *N* is the number of data points.
