# Supplementary material for: Enhanced Adsorptive Properties and Pseudocapacitance of Flexible Polyaniline-Activated Carbon Cloth Composites Synthesized Electrochemically in a Filter-Press Cell
Source: Materials (Basel). 2019 Aug 7;12(16):2516. doi: 10.3390/ma12162516 (PMC6719905; doi:10.3390/ma12162516)
Supplement: Supplementary file 1 [file materials-12-02516-s001.pdf]

materials

# Supplementary Information

## Enhanced adsorptive properties and pseudocapacitance of hybrid Polyaniline-activated carbon cloth composites synthesized electrochemically in a filter-press cell

**César Quijada [1,*], Larissa Leite Rosa [1], Raúl Berenguer [2] and Eva Bou-Belda [1]**

[1] Departamento de Ingeniería Textil y Papelera, Universitat Politècnica de València. Pza Ferrándiz y Carbonell, E-03801 Alcoy (Alicante). Spain.
[2] Instituto Universitario de Materiales, Departamento de Química Física. Universidad de Alicante (UA). Apartado 99. E-03080 Alicante (Spain).
* Correspondence: cquijada@txp.upv.es; Tel.: +34-966-528-419

**Table S1.** Elemental surface composition (in at.%) of a fresh activated carbon cloth and hybrid PAni-ACC composite materials formed by galvanostatic electropolymerization at different electrolysis time.

| Time (min) | %C | %O | %N | %S |
|---|---|---|---|---|
| 0 [1] | 90.9 | 7.7 | 1.2 | 0.2 |
| Side in contact to anode | | | | |
| 10 | 80.4 | 14.6 | 2.4 | 2.6 |
| 20 | 80.1 | 14.1 | 3.0 | 2.8 |
| 40 | 81.7 | 13.2 | 2.9 | 2.2 |
| 60 | 83.8 | 11.1 | 3.0 | 2.2 |
| 120 | 68.2 | 16.5 | 9.4 | 5.9 |
| Side exposed to electrolyte | | | | |
| 10 | 84.3 | 10.4 | 1.9 | 2.6 |
| 20 | 82.9 | 11.7 | 2.8 | 2.6 |
| 40 | 80.9 | 14.2 | 2.7 | 2.2 |
| 60 | 83.8 | 11.9 | 2.0 | 2.4 |
| 120 | 71.2 | 15.5 | 7.7 | 5.6 |

[1] Average of analyses conducted on both sides of the fabric.

**Table S2.** C1s and N1s peak positions and relative abundance (in at.%) after peak fitting.

| Time (min) | C1 (–C=C–/–C–C–) | | C2 (C–O/C–N/C=N/C=N⁺) | | C3 (C=O/C–N⁺) | | | |
|---|---|---|---|---|---|---|---|---|
| | BE (eV) | % | BE (eV) | % | BE (eV) | % | | |
| 0 | 284.8 | 83.2 | 286.4 | 14.0 | 288.2 | 2.9 | | |
| 10 | 284.5 | 71.3 | 285.7 | 23.1 | 287.9 | 5.6 | | |
| 20 | 284.5 | 66.1 | 285.7 | 27.4 | 287.9 | 6.5 | | |
| 40 | 284.6 | 67.9 | 285.8 | 25,7 | 288.0 | 6.4 | | |
| 60 | 284.6 | 68.6 | 285.6 | 25,5 | 287.8 | 5.9 | | |
| 120 | 284.7 | 62.7 | 286.0 | 27.6 | 287.7 | 9.7 | | |
| | **N1** (pyridine, =N–) | | **N2** (pyrrolic/pyridone, –NH–) | | **N3** (quaternary N, N⁺ delocalized) | | **N4** (pyridinic N-oxide, N⁺ localized) | |
| | BE (eV) | % | BE (eV) | % | BE (eV) | % | BE (eV) | % |
| 0 | 398.1 | 23.4 | 399.9 | 25.8 | 400.7 | 41.9 | 401.7 | 8.9 |
| 10 | 398.1 | 6.2 | 399.6 | 28.8 | 400.8 | 46.2 | 402.1 | 18.8 |
| 20 | 398.4 | 4.9 | 399.7 | 31.2 | 400.6 | 50.3 | 402.2 | 13.5 |

| | | | | | | | | |
|---|---|---|---|---|---|---|---|---|
| 40 | 398.2 | 10.2 | 399.7 | 34.6 | 400.8 | 34.2 | 402.3 | 21.0 |
| 60 | 398.4 | 13.4 | 399.8 | 29.4 | 400.5 | 34.8 | 401.8 | 22.4 |
| 120 | 398.3 | 4.0 | 399.4 | 40.1 | 400.2 | 49.3 | 401.9 | 6.6 |

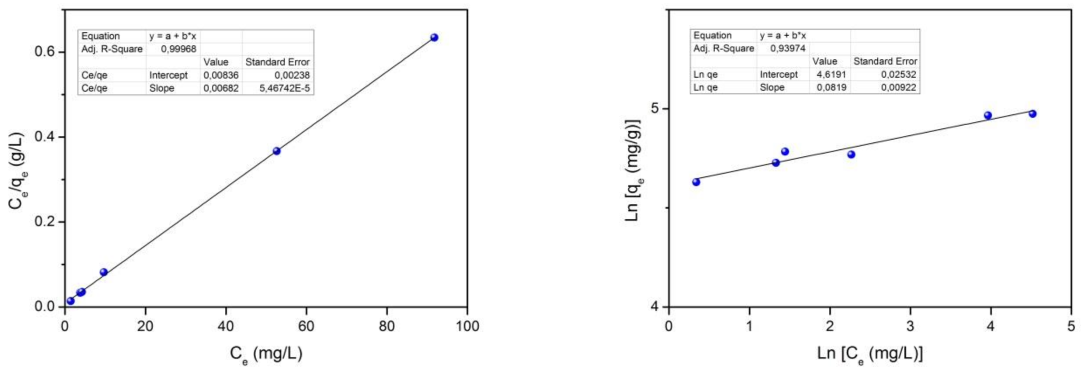


(**a**) (**b**)

**Figure S1.** Isotherm linearized fittings for Acid Red 27 adsorbed on activated carbon cloth (adsorbent dose: 0.04 g, liquid phase volume: 50 mL, temperature: 25 ºC) (**a**) Langmuir isotherm; (**b**) Freundlich isotherm.

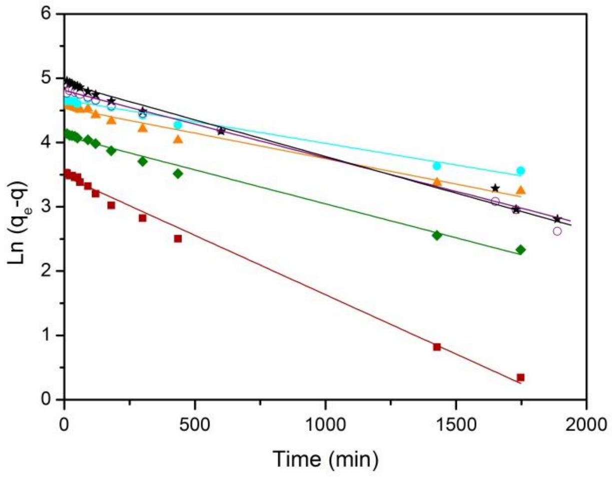


**Figure S2.** Pseudo-first order ln ($q_e$–$q$) vs. $t$ plots for the adsorption of Acid Red 27 on activated carbon cloth at different initial dye concentrations: 25 mg/L (■), 50 mg/L (◆), 75 mg/L (▲), 100 mg/L (●), 150 mg/L (○), 200 mg/L (★); adsorbent dose: 0.04 g, liquid phase volume: 50 mL, temperature: 25 ºC.

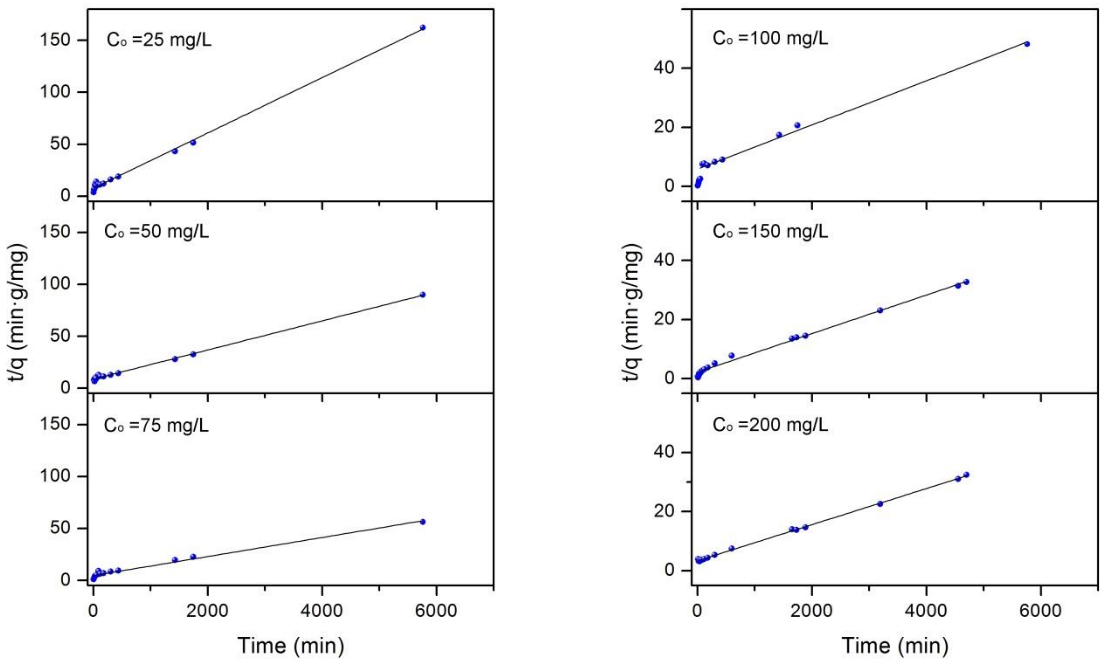


**Figure S3.** Pseudo-second order *t*/*q* vs. *t* plots for the adsorption of Acid Red 27 on activated carbon cloth at different initial dye concentrations; adsorbent dose: 0.04 g, liquid phase volume: 50 mL, temperature: 25 ºC.

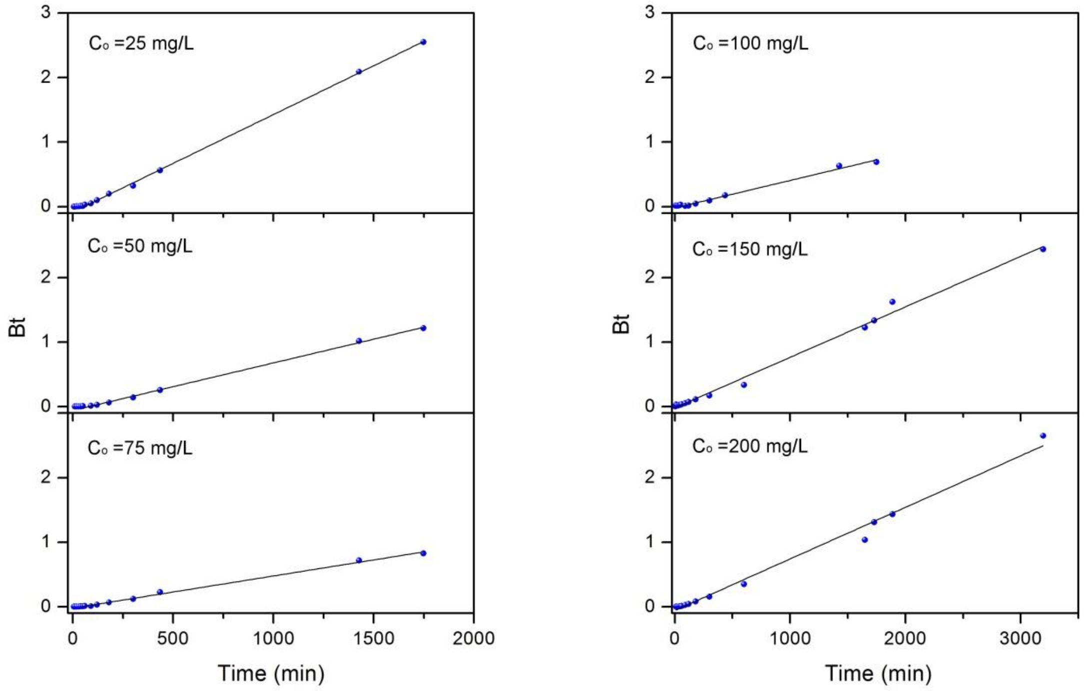


**Figure S4.** Boyd plots for the adsorption of Acid Red 27 on activated carbon cloth at different initial dye concentrations; adsorbent dose: 0.04 g, liquid phase volume: 50 mL, temperature: 25 ºC.

**Table S3.** Boyd plot parameters for the adsorption of Acid Red 27 on ACC ($C_0$: mg·L$^{-1}$; $B$: min$^{-1}$; $D_e$: m$^2$ s$^{-1}$).

| Co | B (×10³) | Y-intercept | R² | Dₑ (×10¹⁷) |
|---|---|---|---|---|
| 25 | 1.51 | –0.084 | 0.999 | 6.34 |
| 50 | 0.74 | –0.066 | 0.999 | 3.14 |
| 75 | 0.52 | –0.025 | 0.998 | 2.20 |
| 100 | 0.43 | –0.027 | 0.993 | 1.82 |
| 150 | 0.78 | –0.017 | 0.994 | 3.30 |
| 200 | 0.80 | –0.057 | 0.990 | 3.38 |

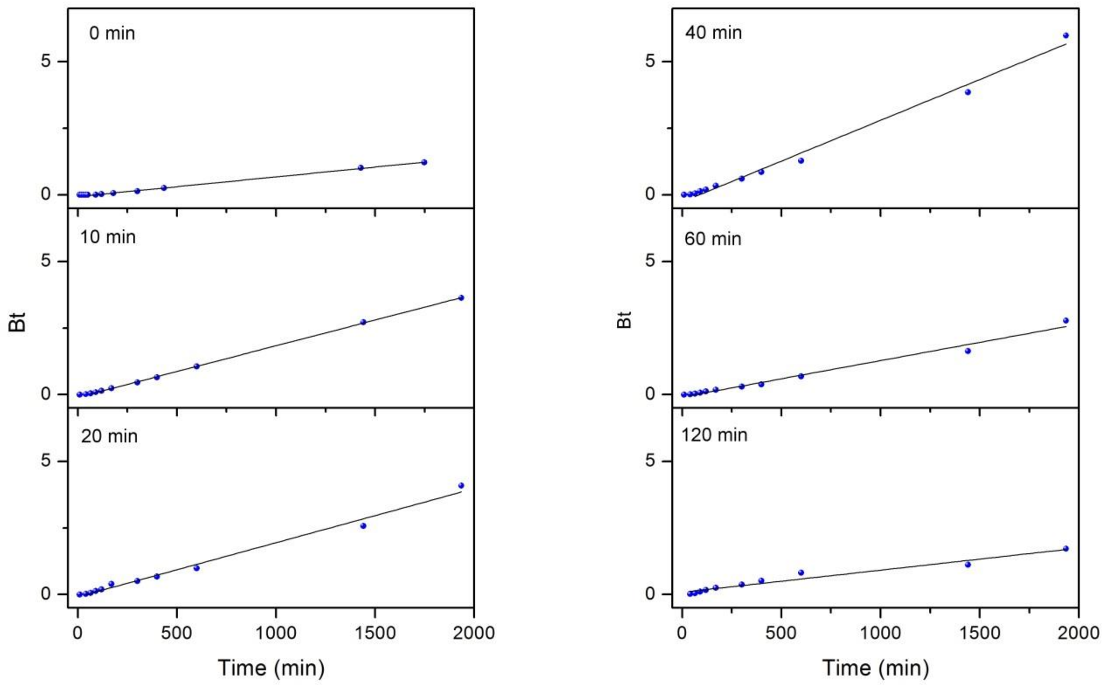


**Figure S5.** Boyd plots for the adsorption of Acid Red 27 on hybrid PAni-ACC composites formed at different electropolymerization time; initial dye concentrations: 50 mg L$^{-1}$; adsorbent dose: 0.04 g, liquid phase volume: 50 mL, temperature: 25 ºC.

**Table S4.** Boyd plot parameters for the adsorption of Acid Red 27 (50 mg L$^{-1}$) on hybrid PAni-ACC composites synthesized at different electropolymerization time (time: min; $B$: min$^{-1}$; $D_e$: m$^2$ s$^{-1}$).

| Time | B (×10³) | Y-intercept | R² | Dₑ (×10¹⁷) |
|---|---|---|---|---|
| 0 | 0.74 | –0.066 | 0.999 | 3.14 |
| 10 | 1.93 | –0.096 | 0.999 | 8.16 |
| 20 | 2.04 | –0.087 | 0.989 | 8.59 |
| 40 | 3.06 | –0.262 | 0.990 | 12.9 |
| 60 | 1.37 | –0.086 | 0.983 | 5.77 |
| 120 | 0.08 | 0.087 | 0.957 | – |
